# Innate Immune-Enhancing Effect of *Pinus densiflora* Pollen Extract via NF-κB Pathway Activation

**DOI:** 10.4014/jmb.2309.09026

**Published:** 2023-12-01

**Authors:** Sehyeon Jang, San Kim, Se Jeong Kim, Jun Young Kim, Da Hye Gu, Bo Ram So, Jung A Ryu, Jeong Min Park, Sung Ran Yoon, Sung Keun Jung

**Affiliations:** 1 School of Food Science and Biotechnology, Kyungpook National University, Daegu 41566, Republic of Korea; 2 Division of Agricultural Environment Research, Gyeongsangbuk-do Agricultural Research & Extension services, Daegu 41404, Republic of Korea; 3 COSMAX NBT, INC., Seongnam 13486, Republic of Korea; 4 Research Institute of Tailored Food Technology, Kyungpook National University, Daegu 41566, Republic of Korea

**Keywords:** Cytokines, health functional food, immune enhancement, innate immunity, nuclear factor-kappa B, *Pinus densiflora* pollen

## Abstract

Considering the emergence of various infectious diseases, including the coronavirus disease 2019 (COVID-19), people's attention has shifted towards immune health. Consequently, immune-enhancing functional foods have been increasingly consumed. Hence, developing new immune-enhancing functional food products is needed. *Pinus densiflora* pollen can be collected from the male red pine tree, which is commonly found in Korea. *P. densiflora* pollen extract (PDE), obtained by water extraction, contained polyphenols (216.29 ± 0.22 mg GAE/100 g) and flavonoids (35.14 ± 0.04 mg CE/100 g). PDE significantly increased the production of nitric oxide (NO) and reactive oxygen species (ROS) but, did not exhibit cytotoxicity in RAW 264.7 cells. Western blot results indicated that PDE induced the expression of inducible nitric oxide synthase (iNOS) and cyclooxygenase (COX)-2. PDE also significantly increased the mRNA and protein levels of cytokines and the phosphorylation of IKKα/β and p65, as well as the activation and degradation of IκBα. Additionally, western blot analysis of cytosolic and nuclear fractions and immunofluorescence assay confirmed that the translocation of p65 to the nucleus after PDE treatment. These results confirmed that PDE increases the production of cytokines, NO, and ROS by activating NF-κB. Therefore, PDE is a promising nutraceutical candidate for immune-enhancing functional foods.

## Introduction

The global pandemic of coronavirus disease (COVID-19) has led to a surge in interest in health functional foods that can help to lower the risk of infections. Additionally, as the world’s population ages and the need for a healthy lifestyle increases, the market for health functional foods has significant grown. Especially in the United States and Europe, the supply of immune-boosting products faced unprecedented difficulties resulting from panic buying during the COVID-19 crisis in 2020 [1]. Antiviral vaccines also face challenges such as rapid mutation, the relatively short duration of vaccine efficacy (approximately 6 months), and the risks associated with vulnerable populations. Given these challenges, prevention against infectious diseases through the intake of immune-enhancing functional foods, which do not carry these risk burdens, has become essential [2].

Strengthening innate immunity, which serves as the initial defense mechanism, is crucial to protect our bodies against acute infectious diseases such as COVID-19. Deficiencies in the innate immune response can lead to various diseases, including Sandhoff disease, a brain disorder, and acute respiratory distress syndrome, a severe respiratory illness [3]. Cells involved in innate immunity include macrophages, dendritic cells, natural killer cells, and innate lymphoid cells [4]. In particular, macrophages differentiate into M1 phenotype upon infection by bacteria, viruses, or parasites, producing antimicrobial substances such as nitric oxide (NO) and reactive oxygen species (ROS) to directly eliminate pathogens [5]. They also promote the maturation and activation of adaptive immunity, represented by B and T cells, by producing signaling mediators such as cytokines [5, 6]. Therefore, maintaining the populations and function of macrophages is one of the effective strategies to keep the body’s immune homeostasis.

Pattern recognition receptors (PRRs) located on the macrophage’s cell membrane are crucial in recognizing foreign invading molecules known as pathogen associated molecular patterns (PAMPs), leading to the initiation of various immune responses [7]. After the onset of immune response by PRRs, immune response signaling pathways are constantly activated through nuclear factor kappa B (NF-κB). NF-κB, a major signaling factor involved in receptor-mediated immune responses, serves as a key transcription factor for the synthesis of inducible nitric oxide synthase (iNOS) and cytokines, and helps regulate various diseases [8, 9]. It exists as a dimer composed of p65 and p50 subunits and remains in an inactive state by binding to IκBα, which acts as an inhibitor of NF-κB. However, when IKKα/β is activated by external stimuli, IκBα undergoes phosphorylation, leading to IκBα degradation. Consequently, NF-κB is translocated freely into the nucleus, where it binds to specific promoters and facilitates the expression of the corresponding genes [10]. It also has been reported that the NF-κB pathway plays a notable role in the immune activity of various food materials [11, 12]. Therefore, NF-κB activation could contribute to enhancing innate immunity by macrophages.

*Pinus densiflora*, also called Korean red pine, is a native variety in Korea, constituting approximately 67% of the coniferous forests in the country [13]. The *P. densiflora* pollen, which is collected from male pine trees during spring, contains abscisic acid and *p*-coumaric acid esters [14, 15]. Its cell wall is primarily composed of arabinogalactan and arabinogalactan proteins, which consist of glucuronic acid [16]. Notably, *P. densiflora* pollen exhibits antinociceptive and anti-inflammatory effects [17]. While a previous study has investigated that *Pinus massionians* pollen, which is derived from China, improves the intestinal mucosal immunity of chickens [18], research focusing on enhancing the innate immune function of *P. densiflora* pollen, a Korean cultivar, remains limited.

This study aimed to evaluate the effects of *P. densiflora* pollen (PDE) on enhancing innate immune function. Briefly, PDE increased the production of NO, cellular and mitochondrial ROS (mtROS), and cytokines in macrophages. Furthermore, PDE-induced NF-κB pathway activation and p65 translocation from the cytoplasm to the nucleus. Results demonstrate that PDE can be a beneficial immune-enhancing material by promoting immune function.

## Materials and Methods

### Materials and Reagents

Dulbecco’s Modified Eagle’s medium (DMEM), fetal bovine serum (FBS), and antibiotics (penicillin/streptomycin solution) were purchased from Thermo Fisher Scientific (USA). Primary antibodies against p-p65 (Ser536), IKKα/β, IκBα, p- IKKα/β (Ser176/180), p- IκBα (Ser32), cyclooxygenase (COX)-2, iNOS, and α/β-Tubulin and lipopolysaccharides (LPS) from *Escherichia coli* O111:B4 were purchased from Signaling Technology (USA). Primary antibodies against p65 and Lamin B1 were obtained from Abcam (UK). The primary antibody against β-actin was purchased from Santa Cruz Biotech (USA), and secondary antibody against Pierce Goat Anti-Rabbit IgG, (H+L) and Pierce Goat Anti-Mouse IgG, (H+L) were obtained from Thermo Fisher Scientific Inc.

### PDE Preparation

The *P. densiflora* pollen, was purchased from Agricultural Corporation Hannong Co., Ltd. (Republic of Korea). It was collected from pine trees in Uljin, Republic of Korea. This pollen was mixed with deionized water at a 1:30 ratio under 80°C and then extracted the same temperature for 4 h in a shaking water bath (VS-1205SW1, Vision Scientific, Republic of Korea). Next, the extracted mixture was centrifuged at 8,000 rpm for 30 min (CR22N, Eppendorf Himac Technologies Co., Ltd., Japan). The supernatant was concentrated at 40°C using an RKTS-22060-SNN concentrator (Genevac Ltd., USA) and subsequently lyophilized with an FDU-7024 freeze-dryer (Operon Co., Republic of Korea). The yield of the water extraction process was 20.54%. The obtained PDE powder was dissolved in sterile distilled water and used as a sample.

### Total Polyphenol Contents

The total polyphenol content was measured using modified Folin-Ciocâlteu method [19]. Briefly, 0.3 ml of each extract sample solution was mixed with 0.3 ml of 50% Folin-Ciocâlteu phenol reagent (Sigma-Aldrich Co., USA) and allowed to react at room temperature for 3 min. Then, 0.3 ml of 10% Na_2_CO_3_ solution (Sigma-Aldrich Co.) was added and mixed, followed by reaction at room temperature for 1 h. The absorbance was measured at 700 nm using SPECTROstar^Nano^ (BMG LABTECH, Germany). The content was expressed as milligrams of gallic acid equivalent (GAE) per 100 g of dried sample, with a standard curve constructed using gallic acid (Sigma-Aldrich Co.) dissolved in distilled water at 0-50 μg/ml concentration.

### Total Flavonoid Contents

The total flavonoid content was measured using a modified version of the method described by Yoon *et al*. [20]. We mixed 0.4 ml of the extract solution with 0.12 ml of 0.5% NaNO_2_ solution (Sigma-Aldrich Co.) and allowed this mixture to react for 5 min at room temperature. Then, 0.12 ml of 1% AlCl_3_·6H_2_O solution (Daejung Chemicals Co., Republic of Korea) was added and allowed to react for 3 min. Thereafter, 0.16 ml of 0.5 N NaOH solution (Junsei Chemical Co., Japan) was added and stirred. The absorbance was measured at 510 nm using SPECTROstar^Nano^ (BMG LABTECH). The content was expressed as milligrams of catechin equivalent (CE) per 100 g of dried sample, with a calibration curve constructed using catechin (Sigma-Aldrich Co.) dissolved in distilled water at 0-200 μg/ml concentration.

### Cell Culture

The murine RAW 264.7 macrophage cells were purchased from the Korean Cell Line Research Foundation (Republic of Korea). The RAW 264.7 cells were cultured in DMEM supplemented with 10% FBS and 1% antibiotics (100 U/ml penicillin + 100 μg/ml streptomycin) and incubated in a CO_2_ incubator (Thermo Fisher Scientific) under 37°C, and 5% CO_2_ conditions.

### Cell Viability

The RAW 264.7 cells were seeded at a concentration of 3 × 10^5^ cells/ml in 96 well plates with 100 μl of cell suspension added to each well and cultured in a CO_2_ incubator. After 24 h, 10 μl of 5 mg/ml Thiazolyl Blue Tetrazolium bromide (MTT) reagent (Sigma Aldrich) was added to each well. After 2 h of treatment with MTT reagent, 80 μl of the medium from each well was carefully removed, and 100 μl of Dimethyl sulfoxide (DMSO, Sigma Aldrich) was added to each well. The plates were shaken at 100 rpm for 30 min to ensure complete solubilization of formazan crystals formed by viable cells. Subsequently, the absorbance was measured at wavelength of 550 nm using microplate reader (Bio-Rad Inc., USA).

### Nitrite Assay

The RAW 264.7 cells were seeded at 3 × 10^5^ cells/ml in 96 well plates, with 200 μl of cell suspension added to each well and incubated overnight. RAW 264.7 cells were then treated with different concentrations of PDE (25, 50, and 100 μg/ml) and cultured for 24 h. After the treatment period, 100 μl of cell culture medium from each well was mixed with 100 μl of Griess reagent, which contained a solution of 0.2% N-(1-naphthyl)-ethylenediamine dihydrochloride (NED) combined with 5% phosphoric acid in 1% sulfanilamide in a 1:1 ratio. The mixture was incubated on a shaker at 100 rpm for 30 min and the absorbance of the reaction mixture was measured at 550 nm by using a microplate reader (Bio-Rad Inc.). For nitrite quantification, sodium nitrite was diluted to various concentrations and used to generate a standard curve for nitrite measurements.

### ROS Production

Cellular ROS production in RAW 264.7 cells was assessed by 2',7'-dichlorofluorescein diacetate (DCF-DA) using a fluorescence reader and a fluorescence microscope. The RAW 264.7 cells were seeded at 3 × 10^5^ cells/ml in 96 well plates and incubated overnight. Subsequently, these cells were treated with different PDE concentrations (25, 50, and 100 μg/ml) and cultured for 24 h. After the treatment period, they were washed twice with 200 μl of warm phosphate-buffered saline (PBS). To assess ROS production, we treated the cells with 20 μM DCF-DA in a serum-free medium, followed by incubation for 30 min in a CO_2_ incubator. The dye-containing medium was completely removed, and the cells were washed twice with 200 μl of PBS. The fluorescence intensity of the DCF-DA dye, indicative of ROS production, was measured using a microplate reader (Bio-Rad Inc.) at an excitation wavelength of 485 nm and an emission wavelength of 530 nm. Furthermore, cellular ROS production was visualized using fluorescence microscopy (Leica, Germany) and analyzed using LAS X microscope software (Leica).

### mtROS Staining

The RAW 264.7 cells were seeded in an 8-well chamber (ibidi, GmbH, Germany) at a 1.5 × 10^5^ cells/ml concentration with 200 μl per well and cultured overnight. Subsequently, the cells were treated with PDE at 50 and 100 μg/ml concentrations and incubated for 1 h. After washing twice with warm PBS, the cells were stained with 5 μM MitoSOX Red mitochondrial superoxide indicator (Invitrogen, USA) for 10 min in a CO_2_ incubator according to the manufacturer’s instruction. After washing three times with warm PBS, the cells were fixed with 4% paraformaldehyde. Thereafter, they were again washed three times with warm PBS. The nuclei were stained with 4',6-diamidino-2-phenylindole (DAPI) (VECTASHIELD: Vector Laboratories, USA). The MitoSOX was oxidized by the superoxide produced in mitochondria and indicated red fluorescence, which was visualized using fluorescence microscopy (Leica-Microsystems).

### Enzyme-Linked Immunosorbent assay (ELISA)

The production of cytokines IL-6. IL-1β, and TNF-α was determined by ELISA analysis. The RAW 264.7 cells were seeded at a 1.5 × 10^5^ cells/ml in 96-well plates, with 200 μl of cell suspension added to each well and incubated for 24 h. These cells were then treated with different PDE concentrations (25, 50, and 100 μg/ml). Following a 24 h incubation period, the supernatant from the cell culture medium was collected. The cytokine concentration in the collected supernatant was analyzed using murine IL-6, TNF-α, and IL-1β uncoated ELISA kits (Invitrogen) according to the manufacturer’s instructions.

### Quantitative Reverse Transcription Polymerase Chain Reaction (PCR)

The RAW 264.7 cells were seeded at a 3 × 10^5^ cells/ml in 10 cm dishes and incubated overnight. Following the incubation period, these cells were treated with PDE at 50 and 100 μg/ml concentrations and incubated for 30 min. The total RNA was extracted from cells using RNAiso Plus (Takara Bio Inc., Japan) according to the manucfacturer’s instructions. Subsequently, cDNA was synthesized using the ReverTra Ace qPCR RT Kit & Master Mix (Toyobo, Japan) in accordance with the manufacturer’s instructions. The synthesized cDNA was used for quantitative PCR (qPCR) with SYBR Green Realtime PCR Master Mix (Toyobo) according to the manufacturer’s instructions. The primer sequences are displayed in Table 1 presents the primer sequences. Relative gene expression levels were normalized using glyceraldehyde-3-phosphate dehydrogenase (GAPDH) and the comparative ΔΔCq method using the CFX Maestro Software (Bio-Rad Inc.).

### Western Blot Analysis

The RAW 264.7 cells were seeded at 3 × 10^5^ cells/ml in 6 well plates with 200 μl of cell suspension added to each well and incubated overnight. Following the incubation period, the cells were treated with different PDE concentrations (25, 50, and 100 μg/ml) and cultured for a specific reaction time of either 30 min or 24 h, as indicated. After the treatment period, the cells were washed twice with cold PBS. Subsequently, 200 μl of lysis buffer (Cell Signaling Technologies, USA), supplemented with phosphatase inhibitor cocktail (Thermo Fisher Scientific), was added to each well for cell lysis. To ensure efficient cell lysis, the collected cells were vortexed once every 10 min for a total of 30 min. After 30 min, the collected cell suspension was centrifuged at 4°C, and 13,652 ×*g* for 15 min and then carefully collected. Moreover, protein quantified using a DC protein assay kit reader (Bio-Rad Laboratories, Inc.) according to the manufacturer`s instructions. The subsequent steps of the experimental procedure were conducted on the basis of previously described methodology [21].

### Nucleus and Cytoplasmic Fraction

To investigate the PDE-induced translocation of p65 into the nucleus, the separation of cytoplasmic and nuclear fractions was performed. RAW 264.7 cells were seeded in 6 cm dishes at a 3 × 10^5^ cells/ml, with 5 ml of cell suspension added to each dish, and incubated for 24 h. Subsequently, the cells were treated with different PDE (25, 50, and 100 μg/ml) and cultured for 30 min. After the treatment period, the cells were washed twice with cold PBS. To collect the cells, 1 ml of PBS was added to each dish, and the cells were scraped. The collected cells were subsequently centrifuged at 300 ×*g* for 5 min at 4°C to remove the supernatant. For cytoplasmic fractionation, the cell pellet was resuspended in 100 μl of Cytoplasmic Extraction Reagent I (Thermo Fisher Scientific) and vortexed for 10 min on ice. Then, 5.5 μl of Cytoplasmic Extraction Reagent II was added, followed by vortexing for 1 min on ice. The mixture was vortexed again and centrifuged at 16,000 ×*g* for 5 min at 4°C. The resulting supernatant represented the cytoplasmic fraction. For nuclear fractionation, the remaining pellet was treated with 50 μl of Nuclear Extraction Reagent and vortexed every 10 min for 40 min on ice. After 40 min, the mixture was centrifuged at 16,000 ×*g* for 10 min at 4°C. The resulting supernatant contained the isolated nuclear fraction. Furthermore, the protein content of the cytoplasmic and nuclear fractions was analyzed using the western blot method.

### Immunofluorescence

The RAW 264.7 cells were seeded in an 8-well chamber (ibidi, GmbH, Germany) at a 1 × 10^5^ cells/ml with 200 μl per well and cultured overnight. Subsequently, the cells were treated with PDE at 50 and 100 μg/ml concentrations and incubated for 30 min. After washing with PBS, the cells were fixed with 4% formaldehyde. After 15 min, the cells were washed three times with PBS and permeabilized with cold 100% MeoH at −20°C for 15 min. Next, the cells were blocked with blocking buffer (containing 5% FBS and 0.3% Tween 20) for 1 h and then treated with the primary anti-p65, diluted in an antibody buffer. These cells were incubated with the primary antibody at 4°C overnight. Thereafter, they were incubated with the secondary antibody, Goat Anti-Mouse IgG (H+L), conjugated with Alexa Fluora 594 (Invitrogen) for 1 h. The nuclei were stained with DAPI (VECTASHIELD: Vector Laboratories), and the translocation of NF-κB p65 was visualized using fluorescence microscopy (Leica-Microsystems).

### Statistical Analysis

All experimental results are presented as mean ± standard deviation using GraphPad Prism 9 software (GraphPad, USA). Multiple comparisons between experimental groups were conducted using one-way analysis of variance followed by *t*-tests. When comparing the control group with the experimental groups, *p* < 0.05 was considered statistically significant.

## Results

### Effect of PDE on Nitrite Production and iNOS and COX-2 Expression and Cell Viability in RAW 264.7 Cells and Its Total Polyphenol and Flavonoid Contents

**Fig. 1:**
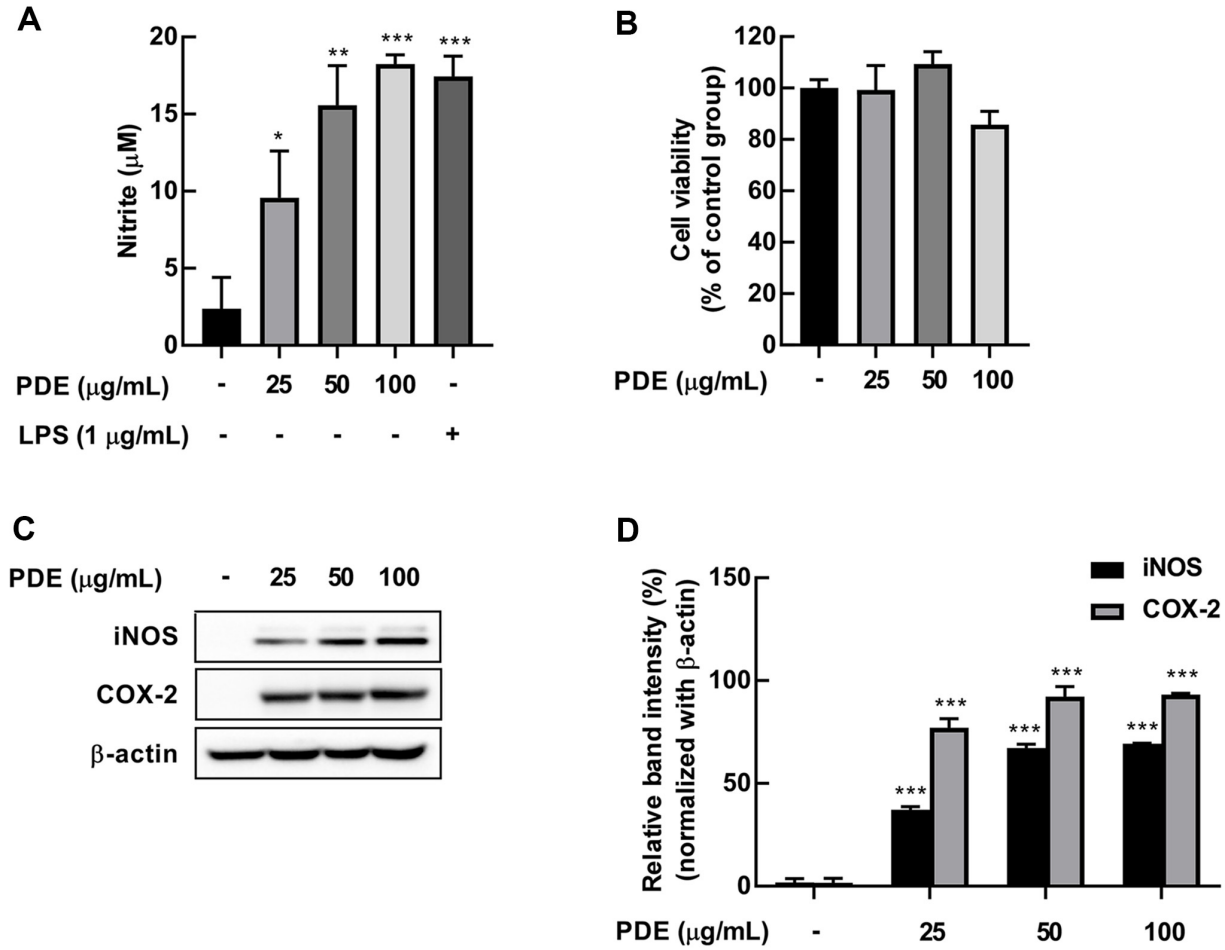
Effects of PDE on nitrite production and expression of iNOS and COX-2 in RAW 264.7 cells. RAW 264.7 cells were treated with PDE at 25, 50, and 100 μg/ml for 24 h. (**A**) PDE significantly induced nitrite production in RAW 264.7 cells. (**B**) PDE did not affect cell viability according to MTT assay. (**C**) PDE significantly induced expression of iNOS and COX- 2 in RAW 264.7 cells. (**D**) Quantification of iNOS and COX-2 expression by PDE. Expression of iNOS, COX-2, and β-actin was detected by Western Blot. The data represent the mean ± SD (*n* = 3). *p < 0.05, **p < 0.01, and ***p < 0.001 vs. control group. PDE, *Pinus densiflora* pollen extract; LPS, lipopolysaccharide; iNOS, inducible nitric oxide synthase; COX-2, cyclooxygenase 2.

Given that NO produced by macrophages helps prevent infectious diseases caused by external pathogens, such as virus and bacteria [22]. we evaluated the effect of PDE as an immune enhancement marker on NO production in RAW 264.7 cells. PDE treatment significantly increased NO production in RAW 264.7 (Fig. 1A) without cytotoxicity (Fig. 1B). Subsequently, the given PDE concentrations (25, 50, and 100 μg/ml) were selected for further experiments to evaluate the immunomodulatory activity of PDE. The iNOS is one of nitric oxide synthesis (NOS) enzymes that catalyzes NO production [23], and COX-2 regulates the production of NO and pro-inflammatory cytokines such as IL-6, IL-1β, and TNF-α to facilitate pathogen clearance during infection [24]. PDE significantly increased the expression of iNOS and COX-2 in RAW 264.7 cells (Fig. 1C and 1D). Polyphenols has immune enhancement capacity by contributing to the prevention of several immune diseases [25]. Therefore, we analyzed the polyphenol and flavonoid contents in PDE. We found that PDE contained 216.29 ± 0.22 mg GAE/ 100 g of polyphenols and 35.14 ± 0.04 mg CE/100 g of flavonoids (Table 2).

### Effect of PDE on ROS Production in RAW 264.7 Cells

**Fig. 2:**
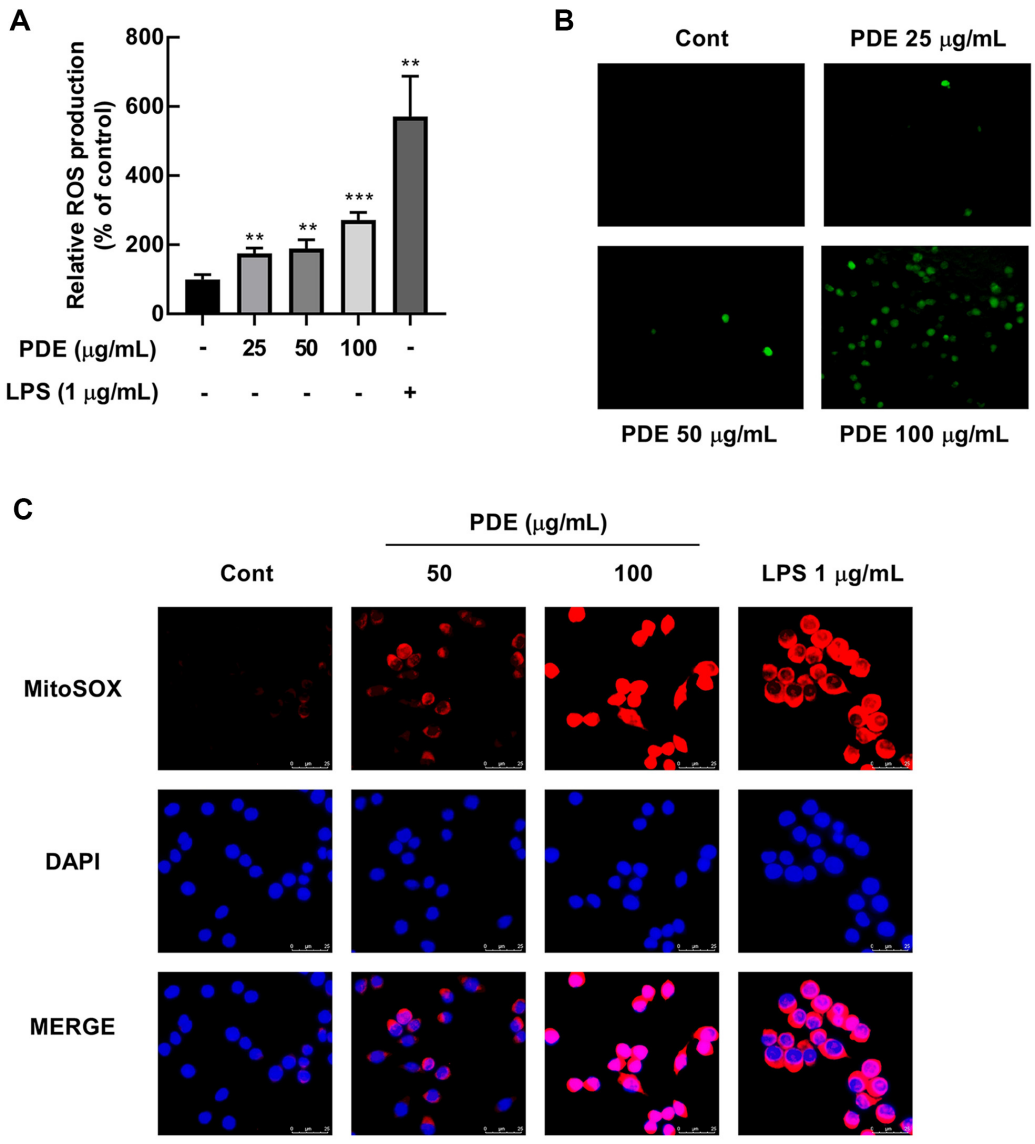
Effects of PDE on ROS production in RAW 264.7 cells. (**A, B**) RAW 264.7 cells were treated with PDE at 25, 50, and 100 μg/ml for 24 h. PDE significantly induced ROS production in RAW 264.7 cells. (**C**) RAW 264.7 cells were treated with PDE at 50 and 100 μg/ml for 24 h. PDE increased the expression of mtROS. Representative images of RAW 264.7 cells stained with MitoSOX Red. The data represent the mean ± SD (*n* = 3). **p < 0.01 and ***p < 0.001 vs. control group. PDE, *Pinus densiflora* pollen extract; LPS, lipopolysaccharide; ROS, reactive oxygen species; Cont, control; MitoSOX, mitochondrial superoxide; DAPI, 4',6-diamidino-2-phenylindole.

ROS produced by macrophages play a crucial role in innate immunity by not only exhibiting antimicrobial activity against invading pathogens but also regulating the production of immunmodulating cytokines [26]. The intracellular production of ROS by PDE was evaluated through DCF-DA fluorescence analysis as the ROS probe. PDE treatment significantly increased the concentration-dependent production of ROS in RAW 264.7 cells (Fig. 2A and 2B). The formation of mtROS formation is closely related to the occurrence of innate immune systems [27]. In addition, PDE treatment increased the expression of MitoSOX, a novel fluorogenic marker for detecting mtROS (Fig. 2C).

### Effect of PDE on Cytokine (IL-6, TNF-α, and IL-1β) Production in RAW 264.7 Cells

**Fig. 3:**
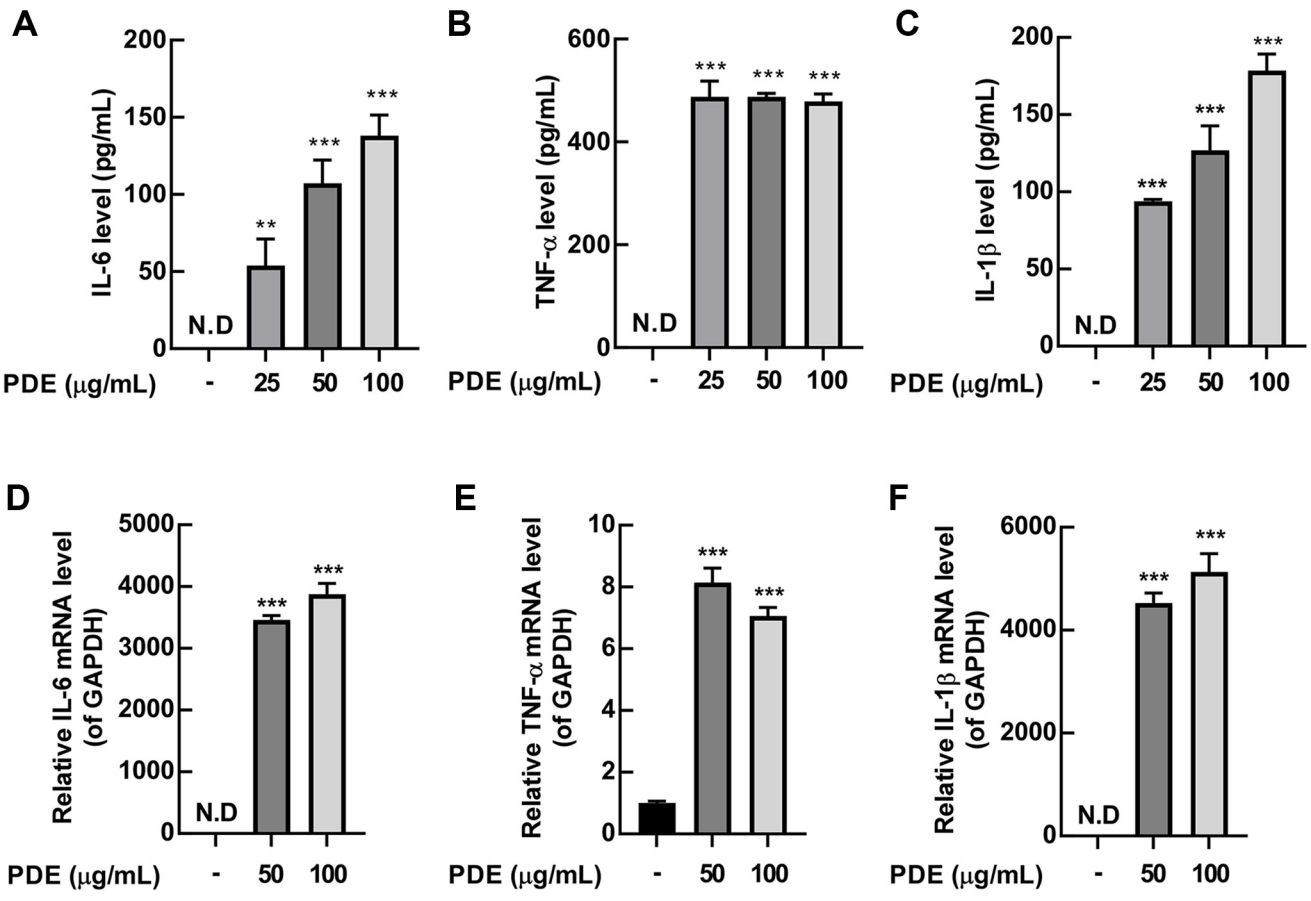
Effects of PDE on the production of IL-6, TNF-α and IL-1β in RAW 264.7 cells. RAW 264.7 cells were treated with PDE at 25, 50, and 100 μg/ml for 24 h. The production of IL-6, TNF-α and IL-1β cytokines was evaluated using ELISA. PDE induced production of IL-6 (**A**), TNF-α (**B**), and IL-1β (**C**) in RAW 264.7 cells culture supernatants. RAW 264.7 cells were treated with PDE at 50 and 100 μg/ml for 24 h. Relative cytokine mRNA level was evaluated by qRT-PCR. PDE increased mRNA expression of IL-6 (**D**), TNF-α (**E**), and IL-1β (**F**) in RAW 264.7 cells. The data represent the mean ± SD (*n* = 3). **p < 0.01 and ***p < 0.001 vs. control group. PDE, *Pinus densiflora* pollen extract; GAPDH, glyceraldehyde-3-phosphate dehydrogenase; N.D., non detection.

Cytokines are low-molecular-weight protein signaling molecules that regulate the activation and growth of immune cells [28]. Cytokines such as TNF-α, IL-6, and IL-1β are widely distributed, playing a central role in host defense. In particular, TNF-α and IL-1β are key cytokines that mediate innate and adaptive immunity [29]. Regarding the effect of PDE on the contents of cytokines released from RAW 264.7 cells, results showed that PDE treatment at various concentrations (25, 50, and 100 μg/ml) significantly increased the production of TNF-α, IL-6, and IL-1β in RAW 264.7 cells (Fig. 3A-3C). Furthermore, to re-verify this result through qRT-PCR assay, we confirmed that PDE increased the mRNA expression of TNF-α, IL-6, and IL-1β (Fig. 3D-3F).

### Effect of PDE on the Activation of NF-κB Signaling Pathway in RAW 264.7 Cells

**Fig. 4:**
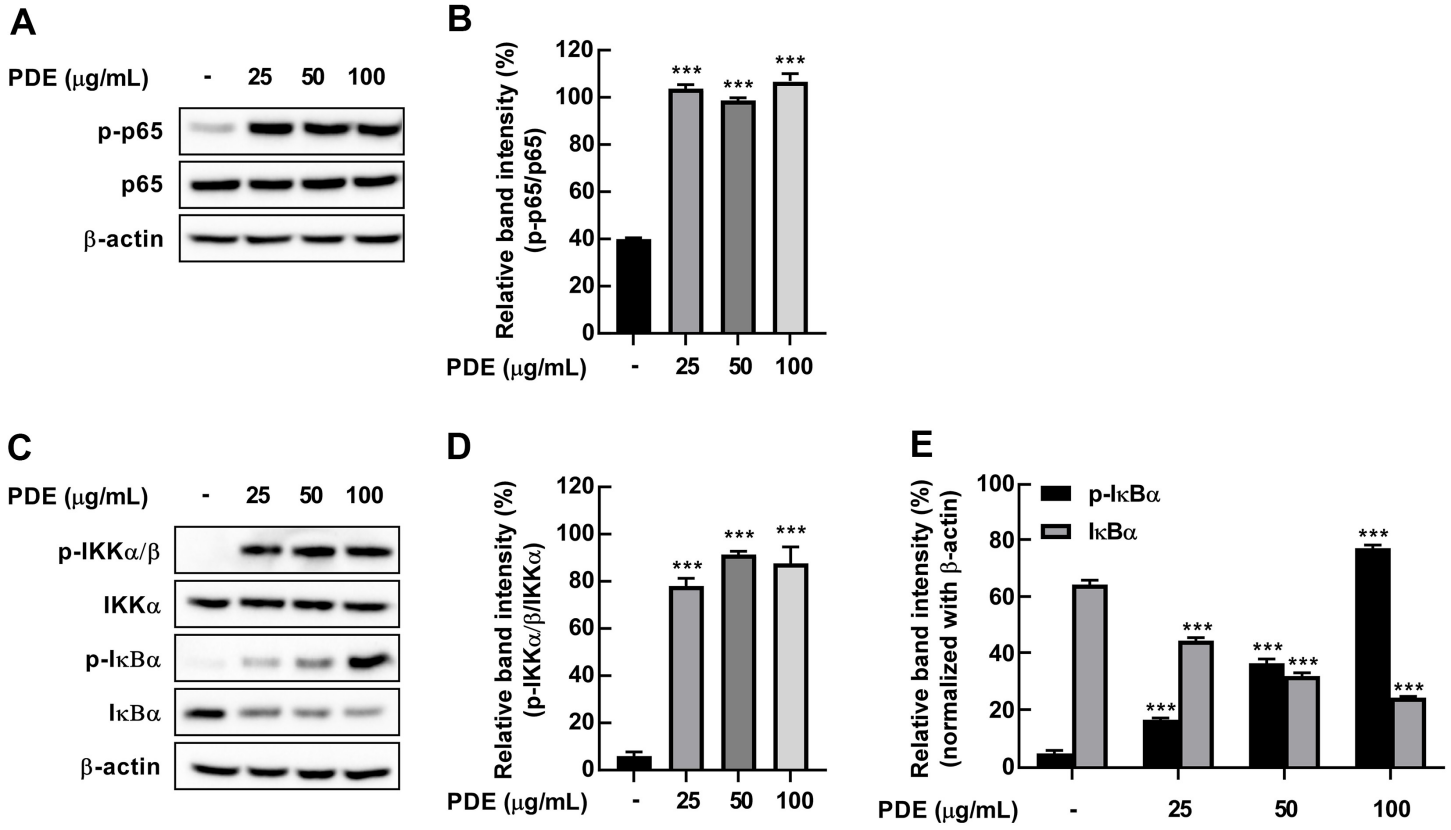
Effects of PDE on the activation of NF-κB signaling pathway in RAW 264.7 cells. RAW 264.7 cells were treated with PDE at 25, 50, and 100 μg/ml for 30 min. (**A**) PDE induced phosphorylation of p65 in RAW 264.7 cells. (**B**) Quantification of PDE-induced p65 phosphorylation. (**C**) PDE significantly induced phosphorylation of IKKα/β and IκBα and IκBα degradation in RAW 264.7 cells. (**D**) Quantification of PDE-induced IKKα/β phosphorylation. (**E**) Quantification of PDE-induced IκBα phosphorylation and degradation. The levels of phosphorylation and expression were detected by western blot. The data represent the mean ± SD (*n* = 3). ***p < 0.001 vs. control group. PDE, *Pinus densiflora* pollen extract.

Previously, we observed that PDE increased production of IL-6, TNF-α, and IL-1β cytokines and NO as an immune-enhancing response. NF-κB is well-known as a key transcription factor involved in the synthesis of iNOS, COX-2, and cytokines [8]. Therefore, we evaluated the effect of PDE on activity on NF-κB p65. PDE significantly increased the phosphorylation of p65 in RAW 264.7 cells at 30 min (Fig. 4A and 4B). Next, we assessed the activity of IκBα and IKKα/β, which are upstream regulators of p65. PDE treatment led to the concentration-dependent degradation and phosphorylation of IκBα (Fig. 4C and 4D). Additionally, PDE induced IKKα/β phosphorylation in RAW 264.7 cells at 30 min (Fig. 4C and 4E).

### Effect of PDE on the Translocation of p65 from Cytosolic to Nuclear Fraction in RAW 264.7 Cells

**Fig. 5:**
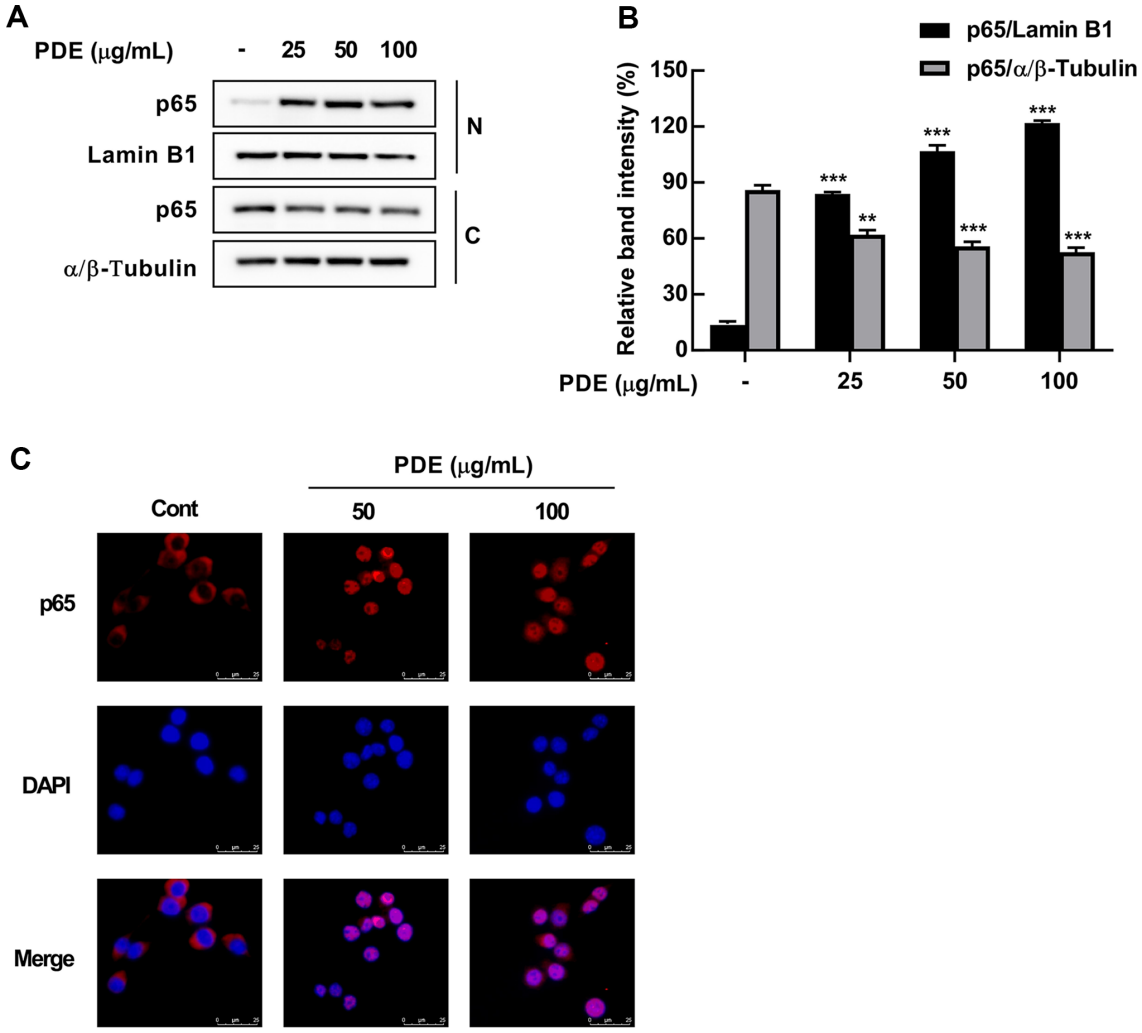
Effects of PDE on p65 translocation in RAW 264.7 cells. RAW 264.7 cells were treated with PDE at 25, 50, and 100 μg/ml for 30 min. (**A**) PDE induced p65 NF-κB nuclear translocation in RAW 264.7 cells. (**B**) Quantification of nuclear p65 and cytosolic p65. The expressions of p65, Lamin B1, and α/β-Tubulin was detected by western blot. RAW 264.7 cells were treated with PDE at 50, and 100 μg/ml for 30 min. (**C**) Immunofluorescence. The data represent the mean ± SD (*n* = 3). **p < 0.01 and ***p < 0.001 vs. control group. PDE, *Pinus densiflora* pollen extract; Cont, control; MitoSOX, mitochondrial superoxide; DAPI, 4',6-diamidino-2-phenylindole.

The phosphorylation of p65 at Ser536 residue and degradation of IκBα lead to the translocation of p65 from the cytosol to the nucleus, where it acts as a transcription factor by binding to specific promoters [30]. To investigate the effect of PDE on the nuclear translocation of p65, we performed cytoplasmic and nuclear fractionation. PDE treatment significantly increased the distribution of p65 in the nuclear fraction while decreasing its distribution in the cytoplasmic fraction (Fig. 5A and 5B). Furthermore, through immunofluorescence analysis, we confirmed that PDE treatment induced the nuclear translocation of p65 (Fig. 5C).

## Discussion

The COVID-19 pandemic has shifted the focus of individuals worldwide toward the central role of immune function in health and well-being. The human body’s immune function is regulated not only by regular lifestyle habits but also by functional ingredients present in the diet; consequently, the trend in developing nutraceutical materials that can modulate immune function has increased [31]. However, the development of materials primarily focusing on anti-inflammatory efficacy prevails, and the materials that exhibit immune-enhancing effects are limited to probiotics, ginseng, and mushrooms, highlighting the lack of options in this regard [32, 33]. Therefore, the development of novel nutraceuticals that can enhance immune function is urgently needed.

Various parts of *P. densiflora* as food ingredients exhibit beneficial physiological activities; related academic research is being conducted. For instance, the bark of *P. densiflora* can lower blood pressure, improve cognitive impairment, protect nerves, and exhibit anti-inflammatory properties [13, 34, 35]. Furthermore, , its leaves have hepatocellular carcinoma prevention and anticancer effects [36, 37]. However, research on the physiological activities of *P. densiflora* pollen remains relatively scarce compared to other parts. Therefore, in this study, we evaluated the immune-enhancing effects of *P. densiflora* pollen in RAW 264.7 cells. Our research findings are expected to play a significant role in elucidating the physiological activities of *P. densiflora* pollen in the academic field.

NO has an antibacterial activity and is widely used as a factor in immune enhancement experiments [22]. However, excessive NO accumulation in the body has been associated with the onset of inflammatory responses [21]. Our results confirmed the intracellular production of NO induced by LPS, a component of the outer membrane of Gram-negative bacteria, that is used as a positive control for immune response and as an inflammation stimulant. We found that the NO production was significantly increased in the PDE-treated group compared with that in the untreated group, but such an increase was lower than that in the LPS-treated group; hence, PDE did not show an excessive inflammatory response. Another important antimicrobial factor is ROS. When infection occurs, ROS generated within the cell permanently oxidize and destroy the cellular structures of external pathogens. ROS are primarily produced by NADPH oxidase (NOX) and mitochondria [38], and notably, mtROS increased by signals from TLR1/2/4 and TRAF6 promote phagocytosis in macrophages [39]. Ruby *et al*., reported that pioglitazone enhances MitoSOX expression in macrophages, aiding in the elimination of pathogen such as *Staphylococcus aureus* [40]. Similarly, PDE increased the production of NO and mtROS as well as cellular ROS. Therefore, PDE could potentially enhance immune function through macrophages activation.

In recent studies, the consumption of foods rich in polyphenols (not only specific polyphenols like resveratrol) has been increasingly associated with an enhanced immune response [41-43]. The total polyphenol content and flavonoid content of PDE were measured as 216.29 ± 0.22 mg GAE/100 g and 35.14 ± 0.04 mg CE/100 g, respectively. These values were approximately 40 and 20 times higher than the total polyphenol content (5.40 mg GAE/100 mg) and flavonoid content (1.79 mg quercetin equivalents/100 mg) of *Azorella compacta* infusion, which possesses immune-stimulating effects such as NK cell and T cell activation [44]. Thus, polyphenols play a pivotal role in driving the immune-enhancing properties of PDE. However, we did not conduct qualitative and quantitative analyses of major phenolic compounds within PDE; hence, further research is needed.

Deficiencies in crucial cytokines, such as IL-6, IL-1β, and TNF-α, have been linked to severe infectious diseases. For instance, compare with C57BL/6J mice IL-6−/− mice exhibit lung macrophage death and inflammatory pneumonia exacerbation induced by *Streptococcus pneumoniae*, as well as allergic asthma [45, 46]. Wada *et al*. conducted research using a TNF-α gene-deficient mouse model produced by gene targeting, and found that TNF-α induced leukocyte recruitment at the infection site and mitigated viral myocarditis progression through the expression of adhesion molecules [47]. In another study by Fremond *et al*., mice with deficient for IL-1 receptor experienced significant weight loss and pulmonary lesions because of impaired innate immune responses following invasion by *Mycobacterium tuberculosis* [48]. Additionally, IL-1β regulates the initiation of Th1 and Th17 adaptive immune responses during infectious conditions [49]. Therefore, the present study evaluated the production of cytokines mediated by PDE. We observed that PDE (100 μg/ml) increased the levels of cytokines IL-6, TNF-α, and IL-1β, which were undetectable in untreated group, to 131.39, 478.97, and 178.5 pg/ml respectively. The polysaccharide from the roots of *Actinidia eriantha* (100 ug/ml), recognized for its immunomodulatory effect, increased IL-6, TNF-α, and IL-1β levels by approximately 24, 12, and 120 pg/ml [50]. In our study, PDE demonstrated significantly potential immune-enhancing effects.

In conclusion, our study confirmed that PDE increases NF-κB activity, leading to the upregulation of iNOS and COX-2 expression, as well as the promotion of NO, ROS, and cytokine production. Therefore, PDE has a promising potential as a food material capable of enhancing the activity of innate immune cells, such as mast cells, thereby demonstrating crucial early defense actions against infections.

## Figures and Tables

**Table 1:** Primer sequences.

Species	Gene	Sense strand (5'-3')	Antisense strand (5'-3')
Mouse	IL-1β	AGT TGA CGG ACC CCA AAA GAT	GTT GAT GTG CTG CTG CGA GA
	IL-6	TGG GAC TGA TGC TGG TGA CAA C	AGC CTC CGA CTT GTG AAG TGG T
	TNF-α	TGG AAC TGG CAG AAG AGG CAC T	AGA GGC TGA GAC ATA GGC ACC G
	GAPDH	ACT CCA CGA CAT ACT CAG C	TCA ACG GCA CAG TCA AGG

**Table 2:** Total polyphenol and flavonoid contents.

	Total polyphenol content (mg GAE/ 100 g)	Total flavonoid content (mg CE/ 100 g)
PDE	216.29 ± 0.22	35.14 ± 0.04

Total polyphenol content is expressed as gallic acid equivalents (GAE). Flavonoid content is expressed as catechin equivalents (CE). Values indicate the average of experiments (*n* = 3) and are represented as mean ± standard deviation.
